# Role of primary care in depression relapse: a qualitative study

**DOI:** 10.3399/BJGP.2024.0384

**Published:** 2025-02-25

**Authors:** Andrew S Moriarty, Emma Williams, Dean McMillan, Simon Gilbody, Carolyn A Chew-Graham

**Affiliations:** Hull York Medical School and Department of Health Sciences, University of York, York.; Hull York Medical School and Department of Health Sciences, University of York, York.; Hull York Medical School and Department of Health Sciences, University of York, York.; Hull York Medical School and Department of Health Sciences, University of York, York.; School of Medicine, Keele University, Keele, UK.

**Keywords:** general practice, primary health care, depression, relapse, follow-up, remission

## Abstract

**Background:**

Relapse contributes to the clinical and societal burden associated with depression. It is not well understood how relapse risk and prevention are managed and discussed between patients and GPs in primary care.

**Aim:**

To understand the extent to which relapse risk and prevention are discussed and managed in general practice.

**Design and setting:**

A qualitative study undertaken in general practice in the UK.

**Method:**

Participants were recruited through general practices. Data were generated using semi-structured interviews and analysed using thematic analysis. Patient and public involvement informed all aspects of the study.

**Results:**

Twenty-three people with lived experience of depression and 22 GPs were interviewed. The following three themes are presented in this paper: perceived determinants of depression course (participants viewed environmental, social, and personal factors as being most important); relapse risk and prevention (relapse was considered important but not consistently or routinely discussed in general practice consultations); and relationships and communication (participants discussed the key role of the GP–patient relationship). Conceptually, relapse was perceived as having limited meaning and usefulness in primary care, owing to the implication of an episodic, discrete course not recognised by many patients and an over-reliance on biomedical diagnosis. Longer-term follow-up and monitoring of depression could be improved in primary care.

**Conclusion:**

We provide an evidence-informed framework to improve practice systems and GP consultations to enhance longer-term care and support for people with depression. Going forwards, acute depression management could be optimised to include discussions of relapse risk and prevention. Brief, scalable relapse prevention interventions are needed for use in primary care.

## Introduction

Relapse of depression is an important and under-researched problem in general practice. The treatment phases of depression are described as: acute phase (aims to improve depressive symptoms and achieve remission), continuation phase (aims to sustain remission to recovery from depressive episode), and maintenance phase (aims to sustain recovery from depressive episode and prevent further episodes).^1^ A significant proportion of people who experience depression and are managed in general practice relapse after the acute phase.^1^^,^^2^ It is unclear to what extent relapse is a concern for people with lived experience of depression and for GPs; how relapse risk is assessed and discussed in practice; and whether relapse prevention is provided in general practice.

Previous research has explored patients’ understanding of relapse and their experiences within primary care.^3^^,^^4^ Fear of depression recurrence has been shown to be a concern for patients, and can cause anxiety and impact on functioning.^5^ A qualitative study, embedded in a trial of relapse prevention,^3^ found that people with recurrent depression often feel disheartened when their GPs advise antidepressant medication in response to relapse or do not show sufficient interest in psychological approaches.^4^ Other research has shown that fear of relapse is a barrier to patients discontinuing antidepressant medication,^6^^–^8 some patients confuse relapse with discontinuation symptoms,^9^ and that patients may not have full confidence in their GP’s ability to discuss antidepressant discontinuation owing to a perceived lack of knowledge and time.^10^ GPs reported that they would be more inclined to continue antidepressant medication in patients with a history of relapse and that time-limited consultations and a lack of evidence-based guidance on long-term depression management result in some patients being suboptimally managed.^10^

This study aimed to explore the perspectives of people with lived experience of depression and of GPs on how relapse is discussed; how risk is assessed; and how ongoing support is provided in primary care.

**Table:** How this fits in

The extent to which relapse of depression is discussed and relapse prevention provided in general practice is not well understood. Findings from this qualitative study suggest that, while relapse is considered an important issue by people with lived experience of depression and GPs, relapse risk and relapse prevention are not routinely discussed in consultations. Ongoing monitoring and longer-term follow-up, beyond the acute phase, are not consistently provided or received. The concept of relapse is of limited usefulness and meaning to both GPs and people with depression; consensus research is required to explore new ways of conceptualising depression relapse and establishing its utility in general practice. We provide a framework for improving longer-term monitoring and follow-up for people with depression in general practice.

## Method

### Qualitative approach

Qualitative methodology was used to understand the meaning underlying participants’ perspectives and experiences. The study was grounded in a critical realist ontology and contextualist epistemology, to acknowledge that there is a reality that we are investigating, but that the truth of that reality is situational and dependent on factors relating to the research methodology used to understand it.^11^^,^^12^ It is reported in line with Standards for Reporting Qualitative Research.^13^

### Researcher characteristics and reflexivity

Reflexivity^14^ was incorporated into this study using a reflexive journal kept by the lead researcher and regular reflective meetings and discussions with the whole research team (Supplementary Information S1). A patient advisory group (PAG) contributed to the following: the research questions and funding application; drafts of materials for ethics application; the co-design of patient information materials; pilot interviews; the co-development of the semi-structured topic guide; and the interpretation of findings.

### Context

**Figure 1.**
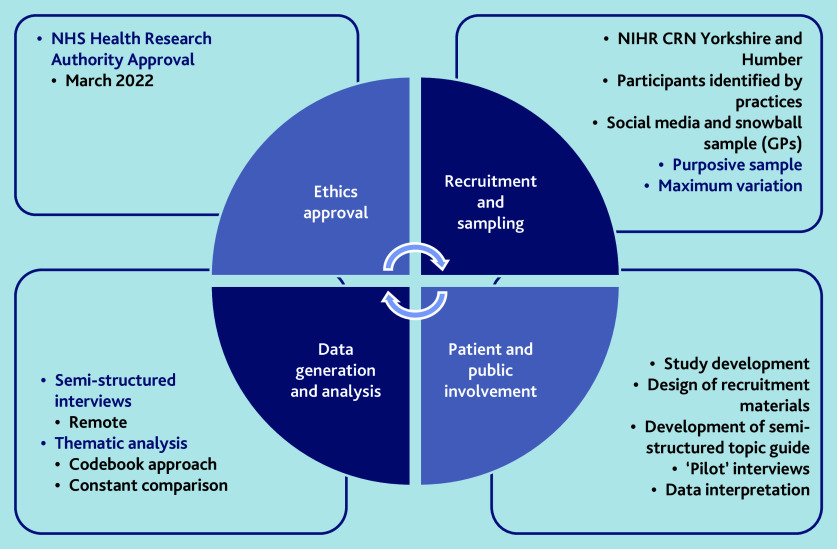
Summary of qualitative methods.

The study was undertaken in general practices in England (Figure 1). Participants were adults (aged ≥18 years) with lived experience of depression (people with comorbid severe mental illness, such as schizophrenia or bipolar disorder, were excluded) and GPs, working within the NHS in any capacity (partner, salaried, or locum).

### Sampling strategy

Purposive sampling was used,^15^ which aimed for maximum variation in the range of factors we considered most likely to have impacted on participants’ experiences. For people with lived experience of depression, these were: gender, age, socioeconomic status (Index of Multiple Deprivation [IMD] of home postcode), and ethnicity. For GPs, these were: years of experience as a GP; contractual status; ethnicity; and socioeconomic status (IMD at practice level). Recruitment was supported by the National Institute for Health and Care Research (NIHR) Clinical Research Network Yorkshire and Humber. Fifteen general practices supported the study.

People with lived experience of depression were recruited through either a search of GP electronic health records followed by an invitation letter, or study advertisement through participating practices. Potential participants were asked to contact the research team directly to express interest in participating. Each general practice was asked to share study information with their GPs and a social media advertisement for GP recruitment was shared on Twitter (now known as X) in April 2022; interested GPs were asked to contact the research team directly. We also incorporated a snowball sampling strategy^15^^,^^16^ for GPs.

We were guided by principles of data saturation^17^ and information power^18^ when determining that a sufficient number of interviews had taken place. Using constant comparison,^19^ we were able to identify the point at which no new themes were apparent on the collection of further data. Our multidisciplinary researcher meetings allowed us to reflect on the richness of the information in the dataset and the extent to which this allowed us to meet the aims and objectives of the study while generating and analysing the data.

### Ethical concerns

Participants were given a minimum of 48 hours with the participant information leaflets, and were given the opportunity to ask questions before signing a consent form. A risk protocol was in place for if the participants disclosed suicidal ideation or intent. All participants were reimbursed for their time in the form of a voucher, in line with NIHR^20^ or British Medical Association guidance.

### Data collection and processing

GPs and people with lived experience of depression were interviewed concurrently, from May–October 2022. Interviews were guided by semi-structured topic guides (Supplementary Information S2), which were modified iteratively as data were collected and analysed. Demographic information was collected to contextualise the data.

Most interviews were conducted using a secure video-conferencing platform (Microsoft Teams); two interviews were conducted by telephone. All interviews were digitally recorded, fully transcribed, and anonymised, and securely stored on a secured university-shared drive. Data will be preserved for 10 years.

### Data analysis

We used a form of codebook thematic analysis (TA),^21^^,^^22^ incorporating principles of constant comparison, wherein new data were systematically compared with previously analysed data.^19^ Analysis was inductive; initial codes were assigned in NVivo 12, without a pre-existing coding framework, and themes were then generated ‘bottom up’ guided by the data. Data from both groups of participants were analysed concurrently and findings were triangulated throughout data analysis. Coding was done by a single researcher, with a random selection of five transcripts (11.1%) coded by two researchers to ensure consistency. The coding and thematic frameworks were regularly reviewed by the whole multidisciplinary team and PAG.

### Techniques to enhance trustworthiness

Trustworthiness^23^ was enhanced through regular discussion and triangulation of findings with the wider multidisciplinary team and PAG. This process ensured the following: an adequate number of interviews; analysis allowed for contradictory accounts; comparisons and contrasts between data from GPs and people with lived experience of depression; and transparency in reporting our approach to data collection and analysis.^24^

## Results

We interviewed 23 people with lived experience of depression and 22 GPs (Table 1).

**Table 1.** Characteristics of participants

**People with lived experience of depression (*n* = 23)**	

**Gender, *n***	
Female	11
Male	12

**Ethnicity, *n***	
White British	21
Asian (Indian) and British mixed	1
British Pakistani	1

**Mean age in years (range)**	45.7 (24–75)

**Socioeconomic status (least deprived to most deprived [IMD])**	1–10

**Mean interview length in minutes (range)**	55.9 (34–80)

**GPs (*n* = 22)**	

**Gender, *n***	
Female	12
Male	10

**Ethnicity, *n***	
White British	15
Asian	6
Black African	1

**Mean age in years (range)**	40.9 (30–61)

**Socioeconomic status (least deprived to most deprived [IMD])**	1–10

**Range of job roles**	
Partner	11
Salaried	10
Locum	1

**Mean years of experience as fully qualified GP (range)**	10.4 (1–30)

**Mean number of clinical GP sessions per week (range)**	5.9 (2–11)

**Mean interview length in minutes (range)**	53.3 (39–76)

*IMD = Index of Multiple Deprivation.*

We generated the following three themes: perceived determinants of depression course; relapse risk and prevention; and relationships and communication. Illustrative examples from the data are presented to support the analysis. Participants are identified by a label indicating: type of participant (P for person with lived experience, or GP); sex (male [M] or female [F]); and age.

### Perceived determinants of depression course

Social, personal, and environmental factors were described by all participants as associated with an unfavourable depression course. A distinction was drawn between ‘external’ factors (social and environmental factors) outside the person’s control, and ‘internal’ factors (personal factors).

External factors included the following: work and financial issues; relationship and family issues; and childhood adversity, trauma, and educational under-performance. Increased social connectedness and support were viewed as protective.

*‘I think the social determinants of health are really important. Things like poverty, lack of work, financial struggles … social networks. If people tend to be in a stable relationship, children, family, community … well ingrained within the community or a faith network … they tend to do better*.’

(GP6, M, 33 years)

People with lived experience of depression mentioned that social support was an important protective factor against relapse. Participants explained the importance of GPs capturing this in a meaningful way in practice.

‘*I think probably that is one of the questions a GP should ask, do you live alone … if they’ve got family nearby … friends, that kind of thing. That is important to find out about people.’*

(P13, F, 57 years)

Internal factors were described as those perceived to be more innate and included genetic factors, biological predisposition, low self-esteem, personality types, and coping styles.

*‘There is an element of hereditary events, I still have the label in my family that, oh, I take after grandma, I’m one of the* [family name] *genes. There’s always a member in the family that’s got the mental illness and I was the one that got that crown … there’s always that element of hereditary, nature versus nurture, isn’t there?’*

(P15, F, 37 years)

In light of this, participants reflected on the challenges of differentiating depression from associated constructs such as emotional distress.

### Relapse risk and prevention

#### Relapse is important but there is limited discussion in general practice

All participants suggested that relapse was important. People with lived experience of depression described their experience of relapse in evocative and emotive ways, frequently using metaphor.

*‘You were virtually on the top of the ladder and then the next day you were down on the bottom rung … and then you struggled … to climb back up the ladder it would take 3 months probably … It was as quick as that.’*

(P3, M, 67 years)

People with lived experience of depression also reflected that they worried about the prospect of relapse even when well, with some expressing hopelessness about the inevitability of experiencing a relapse.

*‘I do* [worry about relapse]*. I know how low I’ve been many times. I know I’ll get there again and I know it will keep happening.’*

(P2, F, 40 years)

Most people with lived experience of depression reported that they had not had a discussion with their GP about their risk of relapsing or about how to prevent this from occurring.

‘*I don’t know if it’s quite a new thing, if they’ve only just realised that people with depression have relapses or anything, because it’s not something that my doctor ever discussed.’*

(P12, M, 39 years)

People with lived experience of depression felt that a discussion about relapse would be beneficial for educating them about their condition and setting expectations around the course of illness. They thought the appropriateness of such a discussion would be dependent on individual preferences and agreement from patients should be sought before entering discussions.

*‘I think it’s patient dependent but for me personally it would have been important … I think that would have definitely been a good conversation to have at a young age. They should have seen that I have a family history of depression and I’m likely going to have it for a long time, so I should be educated on the long-term effects.’*

(P10, M, 29 years)

Some GPs described feeling that they should discuss the chance of relapsing with patients once they had made an improvement in their mood. The main barrier to this was a concern over whether a patient would want to think about becoming unwell again.

‘*They probably don’t particularly want to consider themselves being ill again because they think, oh, if it’s treated and it’s sorted … But … I think it would be a bit remiss of you not to have that discussion really.’*

(GP8, F, 52 years)

Most GPs recognised the benefits of discussing early warning signs of relapse and relapse prevention, but did not incorporate this into their current practice.

*‘Ah, that’s really interesting. That’s not something that I’ve talked to them about really … recognising the early warning signs and getting help early. That’s a really good point, but not something that I do.’*

(GP1, F, 36 years)

#### Relapse, remission, and recovery: interpretation and conceptualisation

Most people with lived experience of depression explained that the episodic nature of depression implicit in the descriptions of relapse, remission, and recovery is not one they recognised from their experience of longer-term, chronic depressive symptoms.

‘*It’s interesting … looking at some of the terminology that you’ve used in some of the questioning about recovery and relapse. I think when I look back at my history, I’m not sure I’ve ever actually recovered, if I’m perfectly honest. I think it’s just been a case of … I’ve got to function again and just get on with it.’*

(P8, M, 57 years)

GPs reported being uncertain about the relevance of these terms to the care of people with depression in primary care.

*‘I use the term “improved” and talk about their feelings, and I look at their function … I think they probably are within the construct the psychologists would use — we just don’t use those phrases.’*

(GP9, M, 45 years)

Finally, there were some negative connotations with the word ‘relapse’ described by both people with lived experience of depression and GPs, which may form a barrier to the routine use of this term in general practice.

*‘I think relapse you associate with like addiction and stuff, don’t you, you really do associate the word relapse with like alcohol and drug addictions*.’

(P18, M, 34 years)

### Relationships and communication

All participants reported that listening and demonstrating empathy is key to a good GP–patient relationship, described the value of continuity of care for depression management, and recognised the time and resource constraints in primary care.

Analysis suggested that acute management of depression is generally consistent. There was a tension around the most appropriate ways to follow-up patients after this phase, between patient-initiated follow-up (patients are advised when and how to follow-up and retain responsibility for initiating this process) and proactive follow-up (the GP retains responsibility for arranging follow-up for patients). GPs generally thought that patient-initiated follow-up was appropriate in the continuation and maintenance phases.

*‘I think you balance that with this sort of idea of patient-initiated follow-up. I am giving some responsibility to them … I tend to give them some ability to come back and safety net that if things are getting worse rather than better, then they would come back sooner.’*

(GP9, M, 45 years)

People with lived experience of depression reported some concerns and previous adverse experiences arising from this approach. They described feeling as though they had been forgotten about or had not felt well-supported.

*‘So, it’s a great idea but a) if you have no initiative because you’re feeling too ill and b) you ring up and they say you can’t have an appointment for 3 weeks or 6 weeks and you can’t have an appointment with the same GP. They’ve put so many barriers in place that keep you away from the GP … by the time they do get to you, any hope of being able to help or be proactive or follow things up or make people feel cared for has gone.’*

(P4, F, 50 years)

When relying on patient-initiated follow-up, GPs reported thinking that communication around how and why to establish follow-up needs to be explicit and understood by the patient. Many of the GPs described being guided by the patient’s preference, indicating the importance of shared decision making.

*‘I think once they reach a period of stability, I tend to put the focus more on them booking their appointments because really by then they’re better, so they should be able to proactively manage their health … but that has to be carefully explained so they understand.’*

(GP17, M, 35 years)

The main way monitoring occurs longer-term (maintenance phase) is through ‘medication reviews’, which take place after a period of time on repeat medication. Some GPs shared uncertainty, or a lack of confidence in their practice systems, that these would occur within the intended timeframe.

*‘Regardless I tend to put a review in … or I tend to limit the amount of prescriptions rather that they can get. Six months, just a bit of a review. Whether that happens or not is another question in practice … But that’s how I tend to try and get them to come back and engage with us again at an appropriate point, trusting that at least at a year that they will come round to a medication review.’*

(GP5, M, 30 years)

People with lived experience of depression reflected on their experiences of medication reviews. These were generally inconsistently provided and perceived as often being superficial, without a meaningful opportunity for patients to discuss their mental health or make informed choices around their medication.

*‘Obviously, every year you have to have your medical review and they just say, “is everything okay?” So, it’s just easier to say, “yeah”, just get all your medication … and they’ve never really discussed my mood or anything.’*

(P2, F, 40 years)

Participants suggested that ongoing care and review of depression (including relapse prevention) should be situated within general practice rather than in other services. GPs agreed that relapse prevention could be situated in primary care but that this would require additional funding and resourcing.

*‘I think you’ve got to make a business case for it, I’m learning this, that if you want GPs to use a finite resource, they will do that if you show them the evidence that that prevents or helps patients in the long-term, and in the end, results in fewer consultations.’*

(GP9, M, 45 years)

All participants recognised that GP time and resource are limited, which can impact on the quality of care provided and patients’ willingness to seek help from their GP. Some felt that the scope of mental health practitioners located in practices could expand to increase capacity and incorporate relapse prevention.

*‘We recognise absolutely it is going to be far more beneficial for our patients being treated by a mental health worker* [who] *works with us* [so] *we know who we can then talk to, because the care is very disjointed currently … having someone that’s employed on a more local footprint, whether that’s practice level or PCN* [primary care network] *… has the potential to be incredibly useful.’*

(GP17, M, 35 years)

## Discussion

### Summary

Participants perceived social, personal, and environmental factors as important determinants of the course of depression. Discussion of relapse risk was recognised to be important by all participants but not routinely discussed in general practice. The terms ‘relapse’, ‘remission’, and ‘recovery’ themselves appear to have limited meaning to GPs or people with lived experience of depression. The importance of the GP–patient relationship and communication (empathy, listening, and continuity of care) were recognised. GPs and people with lived experience of depression reported consistent experiences in the acute phase of depression; experiences of ongoing care and support were more variable. Both participant groups suggested that relapse prevention could and should be embedded within general practice but this would require additional funding and resource.

**Figure 2.**
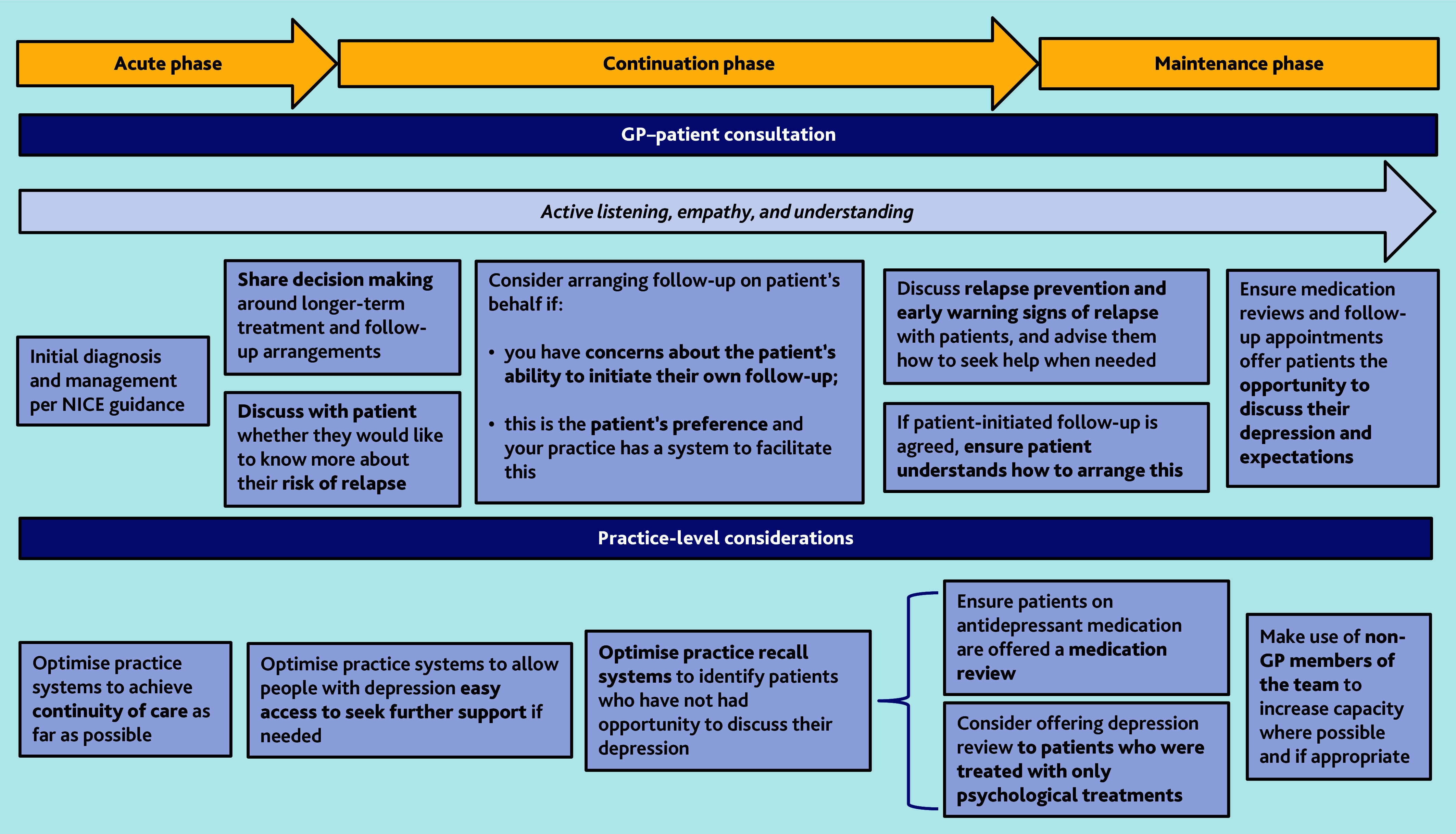
A framework to guide the ongoing care of people with depression in general practice.

We have developed a framework (Figure 2) to guide the ongoing care and follow-up of people with depression in primary care. This framework captures our key findings and provides evidence-informed recommendations around achievable measures that can be taken to improve care in general practice.

### Strengths and limitations

We recruited a good number of participants covering a broad range of demographics in both groups. The inclusion of two groups of participants enabled us to triangulate experiences and perspectives to allow for a deeper analysis and understanding. The sample of people with lived experience had fewer people from minority ethnic groups than we aimed for, despite efforts to increase this through purposive sampling.

A key strength was the involvement of the PAG, who were involved from study conception and helped ensure the findings and implications were relevant and meaningful to people with depression. Owing to COVID-19, interviews were conducted remotely. An advantage of using this approach was that we were able to recruit from a broader geographical area. Evidence suggests that, while there are some minor advantages to in-person interviews, the difference is modest and remote interviews are an acceptable alternative for qualitative health research.^25^^–^27

### Comparison with existing literature

The importance of social, environmental, and personal factors as determinants of depression course is supported by the wider literature. Childhood maltreatment, neuroticism, and rumination have been found to be among the strongest risk factors for relapse of depression.^28^^,^^29^ There is a lack of high-quality evidence exploring the roles of other non-clinical factors in increasing relapse risk.^29^

Our findings suggest that relapse is an important topic for GPs and people with lived experience of depression, but is under-discussed in GP consultations. The importance of relapse to patients has been confirmed in other qualitative research in non-primary care settings.^5^ Most people with lived experience of depression reported not having had a discussion with their GP about relapse risk and prevention, and GPs reported uncertainty and concern as to whether patients want to have this conversation. Similar findings have been reported elsewhere with respect to risk of future depression, which patients were keen to discuss, while GPs thought that patients would not want to know because it could cause anxiety.^30^ When GPs did discuss the risk and depression prevention with patients, reduced incidence of anxiety disorders was reported at 18-month follow-up.^31^

GPs and people with lived experience of depression thought that medication reviews were often superficial or did not occur. This aligns with the literature,^6^^,^^9^ which has shown that patients view clinicians negatively when antidepressant prescriptions are provided without an assessment of need for ongoing treatment.^32^ GPs have previously reported a lack of knowledge of guidance around when and how to stop medication and tended to continue medication, partly owing to concerns around risk of relapse.^6^ There remains some uncertainty regarding best practice around longer-term depression care; statistical risk algorithms for predicting depression outcomes and personalising care are currently of limited accuracy,^33^ and recommendations made in clinical guidelines around depression follow-up are generally based on expert opinion. This may explain some of our findings suggesting inconsistent practice.

Both groups of participants described the importance of relationships, communication, and continuity of care. This is consistent with previous literature highlighting the impact empathy and active listening can have on building trust and rapport within therapeutic relationships.^34^^–^36 Continuity of care is a recognised feature of the GP–patient relationship and its benefits are well-evidenced and uncontroversial.^37^^–^43 The findings of this study reinforce the importance of these elements and the value of the therapeutic consultation in the context of depression.

### Implications for research and practice

The framework presented in Figure 2 captures some of the actions that could be taken to guide quality and care improvement within general practice. Longer-term, undergraduate, and postgraduate curricula should increase focus on the continuation and maintenance phases of depression, in addition to acute diagnosis and treatment. There would be benefit to ensuring that GPs are more aware of risk factors for relapse and how to access relapse prevention for patients where indicated. Clinical education could support primary care clinicians to incorporate simple relapse prevention techniques (such as identifying early warning signs or brief relapse prevention planning) into their routine practice.

A potential facilitator to implementing relapse prevention in primary care is the recruitment of mental health practitioners through the PCN additional roles reimbursement scheme.^44^ Other opportunities for increasing the workforce available for delivering relapse prevention within primary care are through increased integration of health services,^45^ including psychological therapies services. There is potentially a more prominent role for community partners and third-sector organisations in the longer-term support of people with depression. These suggestions would require policy change, local collaboration, and additional primary care resourcing.

These findings suggest we need a new approach to thinking about relapse and longer-term care of depression in primary care. Relapse, recurrence, and associated terms were thought to imply an episodic, discrete disorder, which did not resonate with participants’ experiences, and were overly dependent on a biomedical diagnosis of depression rather than other constructs associated with emotional symptoms (for example, distress).^46^ Some researchers have indeed suggested that depression may require a new classification system and taxonomy for primary care.^47^ Further work to empirically validate these terms and agree useful concepts and outcomes for research and care requires further qualitative work and consensus research with lay representatives, mental health professionals, and primary care experts.

Finally, we recommend a new approach to longer-term depression management. Despite the fact that the vast majority of people with depression are managed in primary care,^48^ evidence for the effectiveness and cost-effectiveness of interventions to prevent relapse is lacking.^49^ Existing strategies that manage depression as a chronic condition (for example, collaborative care,^50^ wherein longer-term care is delivered by a case manager working alongside GPs), could be implemented more widely. Research is needed to guide the optimisation of acute depression management (to incorporate elements that mitigate relapse risk) and the development of scalable relapse prevention interventions, tailored for primary care.

## Data

The dataset is not publicly available, however, anonymised data can be shared with other researchers on reasonable request.
